# Cold-inducible RNA binding protein promotes breast cancer cell malignancy by regulating Cystatin C levels

**DOI:** 10.1261/rna.076422.120

**Published:** 2021-02

**Authors:** Alberto Indacochea, Santiago Guerrero, Macarena Ureña, Ferrán Araujo, Olga Coll, Matilde E. LLeonart, Fátima Gebauer

**Affiliations:** 1 Gene Regulation, Stem Cells and Cancer Programme, Centre for Genomic Regulation (CRG), The Barcelona Institute of Science and Technology, 08003 Barcelona, Spain; 2 Biomedical Research in Cancer Stem Cells, Vall d'Hebron Research Institute (VHIR), 08035 Barcelona, Spain; 3 Spanish Biomedical Research Network Centre in Oncology, CIBERONC, Spain; 4 Universitat Pompeu Fabra (UPF), 08003 Barcelona, Spain

**Keywords:** CIRBP, CST3, breast cancer

## Abstract

Cold-inducible RNA binding protein (CIRBP) is a stress-responsive protein that promotes cancer development and inflammation. Critical to most CIRBP functions is its capacity to bind and posttranscriptionally modulate mRNA. However, a transcriptome-wide analysis of CIRBP mRNA targets in cancer has not yet been performed. Here, we use an ex vivo breast cancer model to identify CIRBP targets and mechanisms. We find that CIRBP transcript levels correlate with breast cancer subtype and are an indicator of luminal A/B prognosis. Accordingly, overexpression of CIRBP in nontumoral MCF-10A cells promotes cell growth and clonogenicity, while depletion of CIRBP from luminal A MCF-7 cells has opposite effects. We use RNA immunoprecipitation followed by high-throughput sequencing (RIP-seq) to identify a set of 204 high confident CIRBP targets in MCF-7 cells. About 10% of these showed complementary changes after CIRBP manipulation in MCF-10A and MCF-7 cells, and were highly interconnected with known breast cancer genes. To test the potential of CIRBP-mediated regulation of these targets in breast cancer development, we focused on *Cystatin C (CST3)*, one of the most highly interconnected genes, encoding a protein that displays tumor suppressive capacities. CST3 depletion restored the effects of CIRBP depletion in MCF-7 cells, indicating that CIRBP functions, at least in part, by down-regulating CST3 levels. Our data provide a resource of CIRBP targets in breast cancer, and identify CST3 as a novel downstream mediator of CIRBP function.

## INTRODUCTION

RNA binding proteins orchestrate posttranscriptional control of gene expression and are emerging as important modulators of cancer progression (Wurth and Gebauer 2015; Pereira et al. 2017; Moore et al. 2018; García-Cárdenas et al. 2019). Cold-inducible RNA binding protein (CIRBP, also termed CIRP and hnRNP A18) is a stress-responsive protein that partially relocates from the nucleus to the cytoplasm under diverse types of stress such as mild hypothermia, UV-irradiation, endoplasmic reticulum (ER) stress or hypoxia (Lujan et al. 2018). In the cytoplasm, CIRBP binds to the coding sequence and 3′-UTRs of target transcripts and promotes or stabilizes mRNA levels. For example, CIRBP promotes telomere maintenance by increasing the levels of TERT mRNA (Zhang et al. 2016), and enhances the tumorigenic properties of cancer cells by promoting the stability of HIF1α and cyclin E mRNAs (Guo et al. 2010; Chang et al. 2016). However, other modes of CIRBP-mediated regulation have also been reported. For instance, CIRBP stimulates global protein synthesis by promoting 4E-BP1 phosphorylation (Artero-Castro et al. 2009; Chang et al. 2016), and has been proposed to stimulate mRNA-specific translation (Yang et al. 2006; Chang et al. 2016), although direct evidence for the latter is still missing. In addition, CIRBP regulates circadian gene expression by promoting alternative polyadenylation (APA) of target transcripts (Morf et al. 2012; Liu et al. 2013). Under UV-irradiation, CIRBP is transiently recruited to sites of DNA damage, where it promotes repair by modulating the association of DNA repair complexes (MRN and ATM kinase) to DNA damage sites (Chen et al. 2018). CIRBP also stimulates the ERK pathway, an event important for bypass of replicative senescence and protection from apoptosis (Sakurai et al. 2006; Artero-Castro et al. 2009). Intriguingly, CIRBP can augment inflammation and induce pyroptosis by a mechanism presumably unrelated to its RNA-binding capacity. Upon hemorrhagic shock and sepsis, CIRBP is released into the circulation where it binds to the TLR4-MD2 receptor of macrophages, leading to release of TNFα, inflammation and tissue injury (Qiang et al. 2013).

Most of the functions mentioned above are consistent with a pro-oncogenic role of CIRBP. Accordingly, although CIRBP null mice display no obvious phenotype under normal laboratory conditions, they show attenuated hepatocarcinogenesis upon thioacetamide treatment and are less susceptible to dextran sodium sulfate-induced intestinal inflammation and tumorigenesis (Sakurai et al. 2006, 2014, 2015; Masuda et al. 2012).

Despite the relevance of CIRBP in tumor progression, no transcriptome-wide attempts have been done to identify CIRBP targets in a cancerous context. Here we use RNA immunoprecipitation followed by high-throughput sequencing (RIP-seq) to identify CIRBP targets. We focus on breast cancer because (i) previous in vitro evidence indicated that CIRBP could modulate the tumoral properties of MDA-MB-231 breast cancer cells (Chang et al. 2016), (ii) immunohistochemistry (IHC) analysis pointed to the presence of CIRBP in the cytoplasm of cells from breast cancer patients (Artero-Castro et al. 2009), and (iii) the large number of samples present in databases compared to other tumor types allows for higher statistical relevance. We first validated breast cancer as a suitable model to study CIRBP-mediated regulation. Indeed, analysis of patient samples indicated that CIRBP mRNA levels correlate with breast tumor subtype and are an indicator of prognosis for the luminal A/B subtype. Depletion of CIRBP from luminal A MCF-7 breast ductal carcinoma cells resulted in decreased proliferation and clonogenicity while, conversely, CIRBP overexpression in nontumoral breast MCF-10A cells increased these traits. RIP-seq from MCF-7 cells identified 204 high-confident targets, 80% of them novel. To identify functionally relevant targets, we used the MCF-7/MCF-10A cell pair and compared transcriptome changes after CIRBP depletion or overexpression, respectively. Of the 204 targets, 22 showed consistent changes in both cell lines. Network analysis indicated that 13 of these targets were highly interconnected with known breast cancer genes. We provide proof-of-principle analysis showing that one of these novel targets, Cystatin C (CST3) is a functionally relevant CIRBP target in breast cancer, as depletion of CST3 abrogates the deleterious effects of CIRBP depletion. These data indicate that CST3 is a novel mediator of CIRBP function in breast cancer.

## RESULTS

### CIRBP is up-regulated in luminal breast cancer and its expression correlates with poor clinical outcome

**FIGURE 1.**
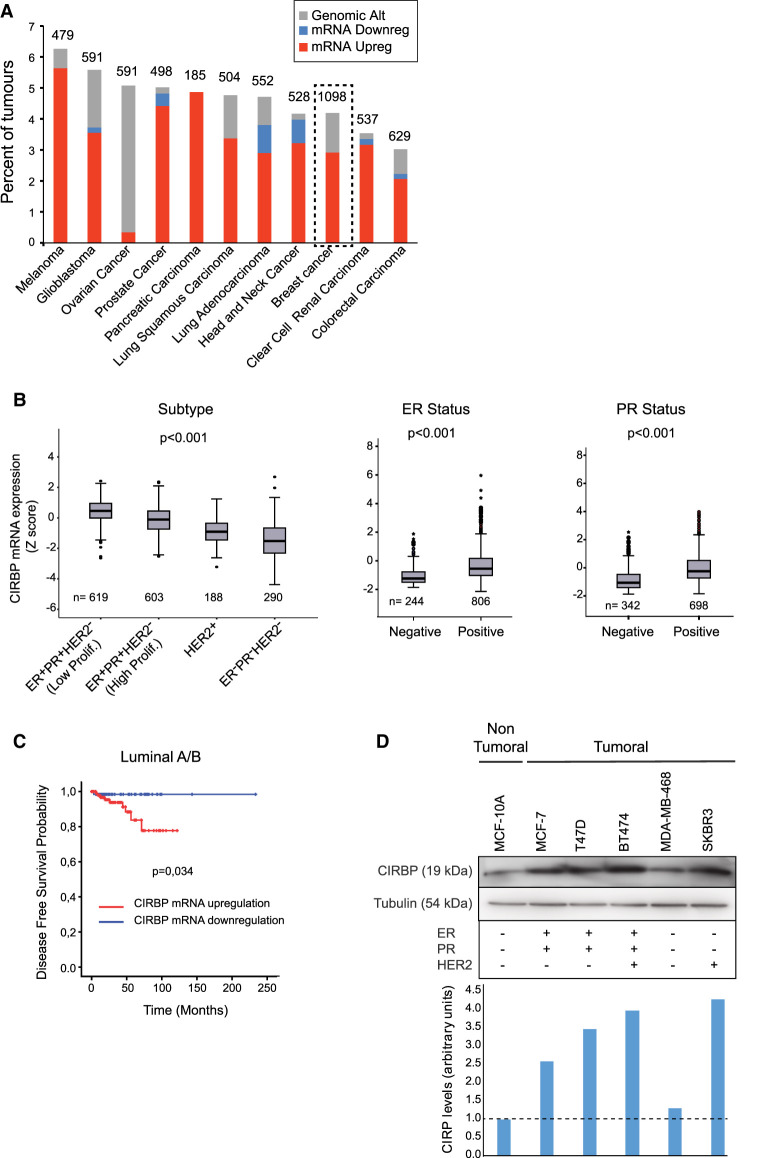
CIRBP is up-regulated in luminal breast cancer and correlates with poor clinical outcome. (*A*) CIRBP genomic alterations (mutations, deletions, and amplifications) and mRNA transcript levels were evaluated across cancer types using the TCGA database. (*B*) Expression of CIRBP mRNA across breast cancer subtypes using METABRIC (*left* panel) and TCGA (*middle* and *right* panels) databases. Box-plot *center* line represents the median, box limits indicate the 25th and 75th percentiles, whiskers extend 1.5 times the interquartile range, and dots represent outliers. Statistics were performed using Kruskal–Wallis (*left* panel) and Mann–Whitney U (*middle* and *right* panels). (*C*) Kaplan-Meier curve for disease free survival in patients with infiltrating ductal carcinoma of the luminal A/B subtype, and negative lymph node. The optimal cut point was found using the Cut-off Finder script for R. Differences between groups was calculated using log rank test. (*D*) Western blot analysis of CIRBP levels in breast cancer cell lines. Tubulin is shown as loading control. The molecular markers of these lines are indicated in the chart *below*. Quantification of the CIRBP signal corrected for tubulin is shown at the *bottom*.

To assess the relevance of CIRBP in cancer, we first interrogated CIRBP expression in patient databases using cBioPortal (https://www.cbioportal.org/) (Cerami et al. 2012; Gao et al. 2013). TCGA database analysis indicated that CIRBP is altered in a variety of human tumors, the most frequent event consisting of mRNA up-regulation (Fig. 1A). We next focused on breast cancer, which had the highest absolute number of CIRBP alterations. Breast cancer is classified in four main subtypes based on the expression of molecular markers, namely estrogen receptor (ER), progesterone receptor (PR) and human epithelial growth factor receptor 2 (HER2) (Harbeck et al. 2019). In increasing order of aggressiveness, tumors are low proliferative ER^+^PR^+^HER2^−^ (luminal A), high proliferative ER^+^PR^+^HER2^−^ (luminal B), HER2^+^ (luminal B and nonluminal) or ER^−^PR^−^HER2^−^ (triple negative). Analysis of CIRBP expression along these subtypes revealed a higher transcript expression in less aggressive hormone-positive (ER^+^PR^+^) breast cancer samples present in TCGA and METABRIC databases (Fig. 1B). Although less aggressive, hormone-positive breast cancer represents the most abundant class (60%–70%), and can metastasize leading to death. To test whether CIRBP levels can function as a prognosis factor, we explored CIRBP transcript prevalence in ER^+^PR^+^ patients with primary infiltrating ductal carcinoma and negative lymph node status. The results indicated that CIRBP up-regulation correlates with poor disease-free survival (Fig. 1C).

In order to select an appropriate cell system to study the function and targets of CIRBP in breast cancer, we next explored CIRBP protein levels in a set of breast cancer cell lines. CIRBP was overexpressed in luminal (MCF-7, T47D, BT474) compared with nonluminal (MDA-MB-468 and SKBR3) and nontumoral (MCF-10A) cells (Fig. 1D). We, thus, selected the luminal MCF-7 and the nontumoral MCF-10A cell line pair for our study.

### CIRBP promotes proliferation

**FIGURE 2.**
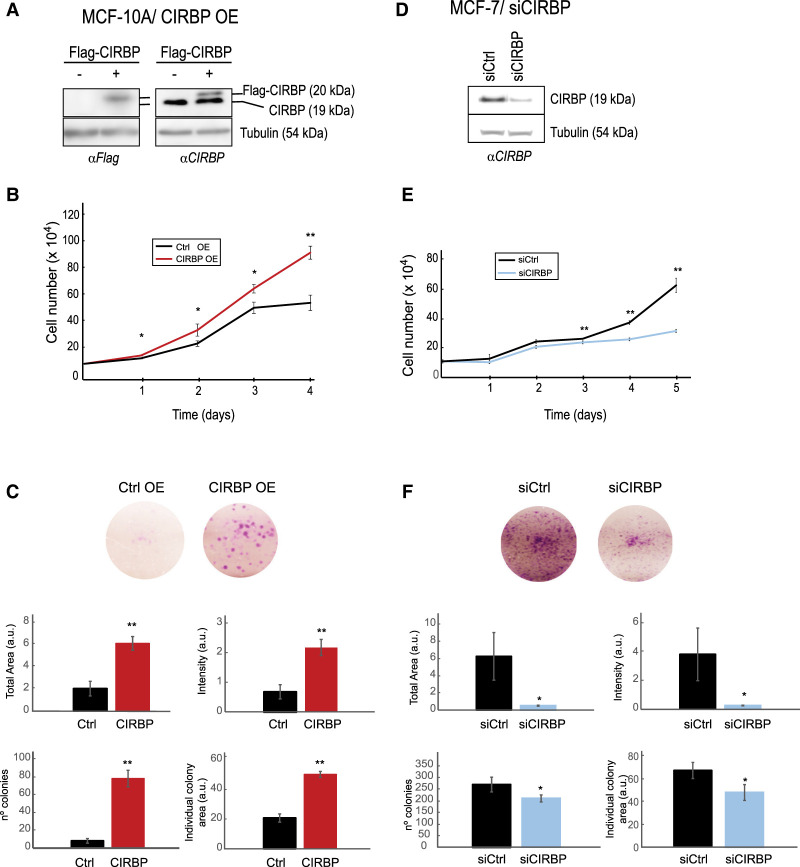
CIRBP promotes tumorigenic traits. (*A*) MCF-10A cells were stably transduced with a retroviral vector expressing CIRBP (+), or with the empty vector (−) as control. Western blots with anti-Flag (*left*) or anti-CIRBP (*right*) antibodies are shown. These cells were tested for proliferation (*B*) and clonogenicity (*C*). (*D*) MCF-7 cells were transiently transfected with siRNA pools against CIRBP or with a control siRNA pool. The efficiency of depletion was assessed by western blot at the beginning of the experiment (day 0). These cells were tested for proliferation (*E*) and clonogenicity (*F*). Error bars represent the standard deviation of triplicate experiments. Statistical significance was assessed by Student's *t*-test (*) *P* < 0.05, (**) *P* < 0.01.

To evaluate the role of CIRBP in our cellular model, we both overexpressed CIRBP in MCF-10A cells and down-regulated CIRBP in MCF-7 cells. Even mild overexpression of CIRBP (to ∼30% of endogenous CIRBP levels) resulted in increased cell proliferation and clonogenicity (Fig. 2A–C), while depletion of CIRBP had the opposite effect (Fig. 2D–F). These results are consistent with a pro-oncogenic role of CIRBP in breast cancer.

### Transcriptome-wide identification of CIRBP targets

**FIGURE 3.**
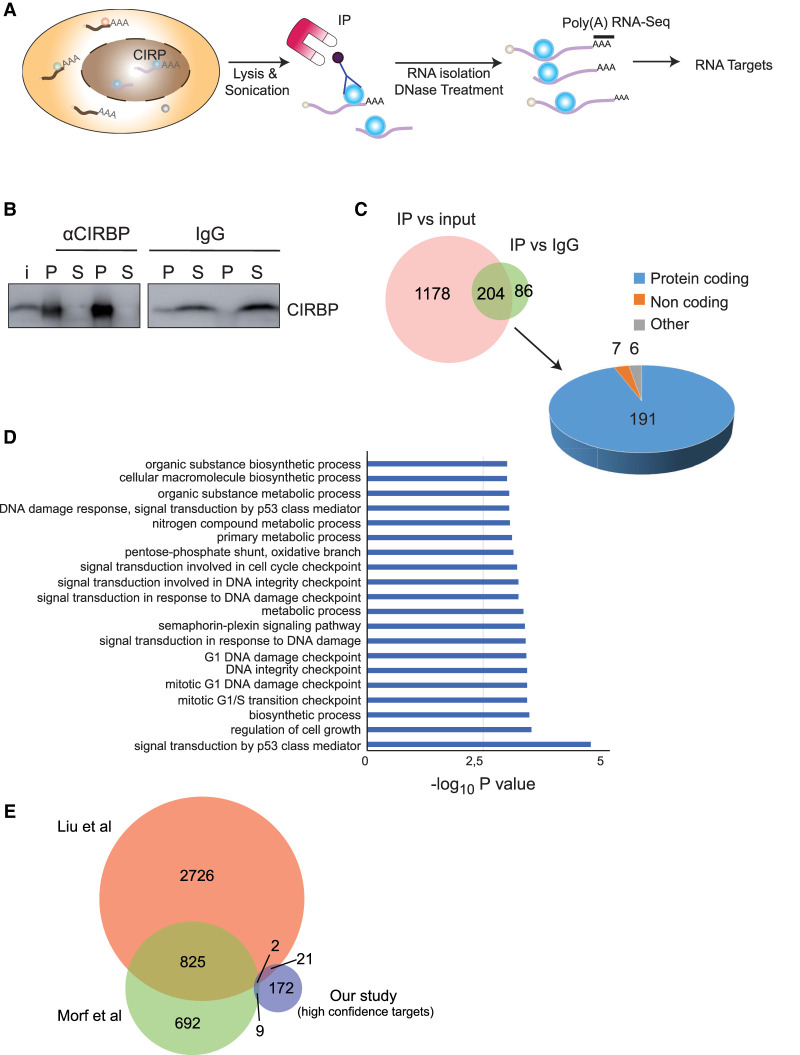
Identification of CIRBP targets. (*A*) Schematic representation of our RIP-seq approach to identify CIRBP targets. (*B*) Efficiency of immunoprecipitation in duplicate experiments. Immunoprecipitations with nonspecific IgG were carried in parallel as control. (i) input, (P) pellet, (S) supernatant. (*C*) A target was considered positive when there was significant enrichment (L2FC > 1, *P*_adj_ < 0.01) in the CIRBP IP versus the input, or in the CIRBP IP versus the IgG control. Targets positive in both analysis (204) were considered high-confident targets. Most of these targets are protein-coding genes (*right*). (*D*) Gene Ontology (GO) analysis of the high confidence targets. The top 20 biological processes were selected for representation. (*E*) Comparison between CIRBP high-confidence targets identified in this study and previous transcriptome-wide analysis of CIRBP targets.

To identify CIRBP mRNA targets in MCF-7 cells in an unbiased, transcriptome-wide manner, we used RNA immunoprecipitation followed by high-throughput sequencing (RIP-seq) (Fig. 3A). Given that CIRBP is located in the nucleus of MCF-7 cells, we chose a cell lysis protocol that included sonication but only mild detergents to preserve RNA–protein interactions. CIRBP was immunoprecipitated from the lysates and the associated RNA sequenced using poly(A) RNA-seq. Parallel reactions with nonspecific rabbit IgG were carried as negative control. The IP efficiency of duplicate experiments is shown in Figure 3B. As expected, only a small proportion of the input RNA was associated to CIRBP (Supplemental Fig. S1A) and the obtained sequences are biased toward the mRNA 3′ end (Supplemental Fig. S1B). We believe that this bias reflects the chosen protocol for the identification of targets rather than an intrinsic property of CIRBP binding, because sonication shears the RNA and poly(A) selection then enriches for 3′ fragments. However, we cannot rule out a preferential binding of CIRBP to 3′-UTRs in MCF-7 cells, as reported in other contexts (Morf et al. 2012; Liu et al. 2013). Sequencing of the two RIP replicates showed a high correlation (Supplemental Fig. S1C). We performed pairwise comparisons of CIRBP IP versus IgG or CIRBP IP versus input, considering either all counts for each gene or only counts in the last three exons to account for the 3′ bias (Supplemental Fig. S1D; Supplemental Table S1). A larger number of targets were retrieved in the CIRBP versus input comparison, while minimal differences were observed using whole gene or last exons counts (Fig. 3C; Supplemental Fig. S1D). Independent RT-qPCR validation of targets selected over a wide range of *P*-values yielded a validation rate of 73% (Supplemental Fig. S1E). We reasoned that robust CIRBP targets should be enriched over both the input and the IgG control; thereby we selected a subset of 204 targets enriched under both comparisons as our high-confident target list (Fig. 3C; Supplemental Table S1). Around 90% of these targets are protein-coding genes while the remaining 10% include long noncoding RNAs and pseudogenes (Fig. 3C; Supplemental Table S1). Gene Ontology (GO) analysis revealed biological processes related with DNA damage and proliferation, in line with previous reports showing that CIRBP is involved in the DNA damage response (Fig. 3D; Yang and Carrier 2001; Yang et al. 2006, 2010; Chen et al. 2018). However, none of our targets had been described in previous CIRBP analyses, suggesting novel mediators for DNA damage regulation by CIRBP. We further compared our list with CIRBP targets defined in two previous transcriptome-wide efforts aimed at the study of circadian gene regulation in the mouse (Morf et al. 2012; Liu et al. 2013). The comparison revealed an overlap of 32 targets (15% of our list) (Fig. 3E). This limited overlap might be due to the different organism (human vs. mice), cell types (epithelial cells vs. fibroblasts), biological contexts (cancer vs. nontumoral) and technologies (RIP vs. CLIP or PAR-CLIP) used in our study compared to previous reports. Altogether, the results indicate that we have identified novel targets of CIRBP in human luminal breast cancer cells.

### CIRBP regulates a network of breast cancer genes

**FIGURE 4.**
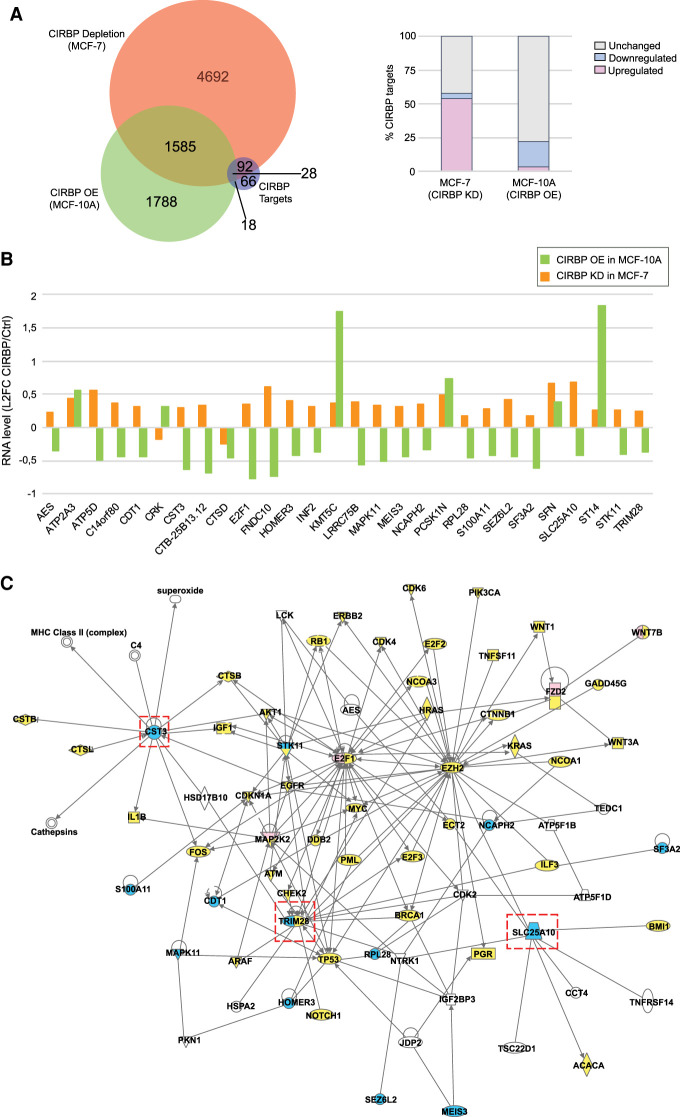
CIRBP regulates a network of breast cancer genes. (*A*) Differences in the transcriptomes of MCF-10A cells overexpressing CIRBP, and MCF-7 cells depleted of CIRBP, with their respective controls were determined by RNA-seq (experiments were performed in duplicate; threshold for significance, *P*_val_ < 0.05). The overlap between genes changing in both cell lines and the CIRBP high-confident target list is shown on the *left*. The direction of change in the high-confident target list is shown on the *right*. (*B*) Detail of the direction and magnitude of change of the 28 CIRBP targets overlapping in *A*. (*C*) Network analysis of CIRBP targets (see text for details). Yellow, known breast cancer gene; blue, CIRBP high-confident target; pink, CIRBP target identified in this study but excluded from the high-confident list. A few targets show two colors, indicative of their classification in two groups. Connections represent interactions at any reported level (i.e., protein–protein, protein–mRNA/DNA, activation); arrows represent activation while bars represent inhibition. Shapes of the nodes: vertical ellipse, transmembrane receptor; horizontal ellipse, transcription regulation; diamond, enzyme; square, cytokine; inverted triangle, kinase; vertical rectangle, G-protein coupled receptor; horizontal rectangle, ligand-dependent nuclear receptor; trapezoid, transporter; simple circle, other; concentric circle, group or complex. Dashed, red squares represent CIRBP targets that are highly interconnected in the network and which were chosen for independent validation.

As mentioned above, CIRBP has been shown to posttranscriptionally regulate the levels of target transcripts. In order to identify functionally relevant CIRBP targets in breast cancer, we evaluated changes in the transcriptomes of MCF-10A and MCF-7 cells after overexpression and depletion of CIRBP, respectively, by RNA-seq analysis (Supplemental Table S2). Most changes observed for the 204 CIRBP targets included up-regulation upon CIRBP depletion in MCF-7 cells or down-regulation in MCF-10A cells upon CIRBP overexpression, indicating a predominant role of CIRBP in mRNA down-regulation (Fig. 4A, right panel). A set of 1613 transcripts changed in both cell types, of which 28 overlapped with our high-confident CIRBP target list (Fig. 4A, left panel; Supplemental Table S2). A close examination of the magnitude and direction of changes in these 28 targets indicated that 22 of them changed in opposite directions in both cell lines, consistent with variations of CIRBP levels (Fig. 4B).

To identify which of these 22 genes was potentially relevant in breast cancer, we performed network analysis. A list of known breast cancer genes was constructed from KEGG (https://www.genome.jp/kegg/), COSMIC (https://cancer.sanger.ac.uk/cosmic) and TCGA databases. This list was intersected with the 22 CIRBP targets using Ingenuity Pathways allowing for direct and up to two indirect interactions. Strikingly, 13 of the 22 targets (59%) were highly interconnected with known breast cancer genes: *CST3, S100A11, MAPK11, STK11, CDT1, HOMER3, TRIM28, RPL28, NCAPH2, SF3A2, SLC25A10, SEZ6L2*, and *MEIS3* (Fig. 4C). Of these, *TRIM28* is a known breast cancer gene (Wei et al. 2016; Czerwińska et al. 2017), while the rest are novel. Of note, four of the cancer genes present in the network (*MAP2K2, E2F1, FZD2*, and *WNT7B*), although absent from our high-confident list of CIRBP targets, are enriched in CIRBP IPs (Supplemental Table S1). These data indicate that our stringent criteria may have resulted in loss of true positives and, together with the high percentage of CIRBP targets included in the network, provides confidence to our selection.

### CIRBP promotes oncogenic traits through regulation of CST3

**FIGURE 5.**
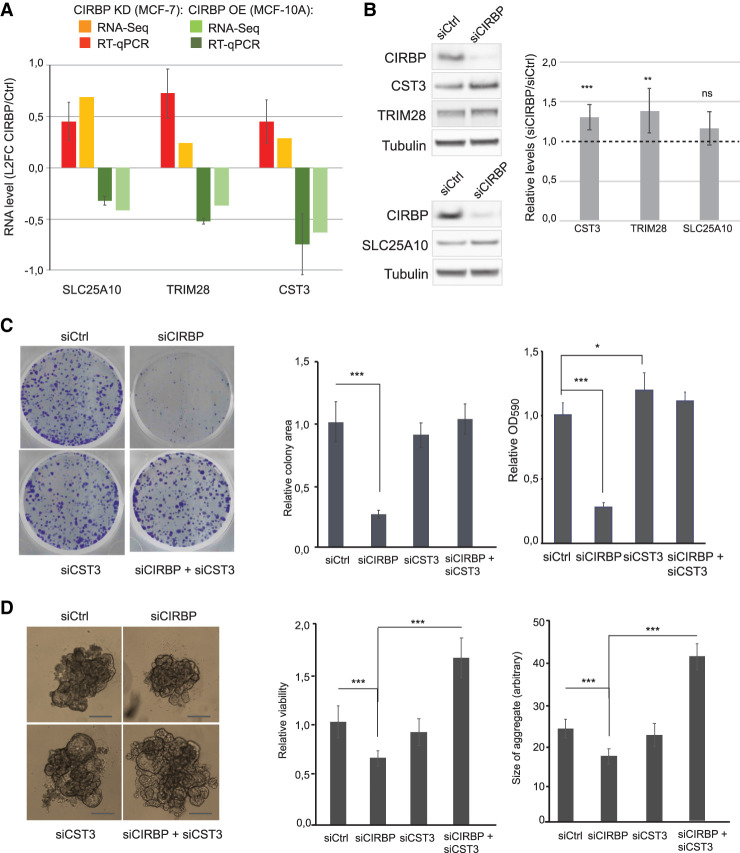
CST3 mediates CIRBP regulation of cancerous traits. (*A*) Validation of RNA-seq results by RT-qPCR of selected targets after manipulation of CIRBP levels in MCF-7 and MCF-10A cells. For comparison, the RNA-seq data are included. Error bars represent the standard deviation of at least two independent experiments. (*B*) Levels of targets in *A* by western blot after depletion of CIRB from MCF-7 cells. Tubulin was used as a loading control. Quantification of at least four experiments, corrected for tubulin and normalized to the siControl (dashed line) is shown on the *right*. (*C*) Clonogenic assay after depletion of CIRBP and/or CST3 from MCF-7 cells. A representative image is shown per condition on the *left*. *Middle* and *right* panels show the quantification of the area of colonies and global plate intensity, respectively. Error bars represent the standard deviation from triplicates carried in parallel. Two independent biological replicates were performed. (*D*) Anchorage independent growth of MCF-7 cells after depletion of CIRBP and/or CST3. Representative images are shown on the *left*. Relative viability and size of aggregates are shown in the *middle* and *right*, respectively. Error bars represent standard deviation (*n* = 6). Significance was determined using Student's *t*-test. (***) *P*_val_ < 0.001, (**) *P*_val_ < 0.01, (*)*P*_val_ < 0.05; (ns) nonsignificant.

To validate CIRBP regulation of select targets, we focused on three of the highly interconnected genes: *CST3, SLC25A10*, and *TRIM28*. We corroborated the RNA-seq results obtained after CIRBP depletion and overexpression by using RT-qPCR (Fig. 5A). Results were further validated by western blot after depletion of CIRBP from MCF-7 cells (Fig. 5B).

As a proof of principle, we chose CST3 for functional studies. Our aim was to test whether CIRBP-mediated regulation of CST3 was relevant to modulate the tumorigenic properties of MCF-7 cells. To that end, and given that CIRBP down-regulates CST3 levels, we tested whether CST3 depletion rescues the phenotype resulting from CIRBP depletion. The results showed that, while CST3 silencing alone had minor effects on MCF-7 clonogenicity, it did overcome the reduced clonogenicity resulting from CIRBP depletion (Fig. 5C, see efficiency of depletion in Supplemental Fig. S2A). Similar results were obtained when MCF-7 cells were grown in nonadherent plates. In these conditions, MCF-7 cells grow as 3D-aggregates whose size and viability decrease after CIRBP knockdown. Concomitant depletion of CST3 eliminates and even surpasses this effect (Fig. 5D, see efficiency of depletion in Supplemental Fig. S2B). These results indicate that down-regulation of CST3 mRNA levels by CIRBP contributes to the oncogenic features of MCF-7 breast cancer cells.

## DISCUSSION

Despite the fact that luminal tumors A and B have a better prognosis (compared to Her2+ and TN), in the last decades an increase in the number of luminal A/B patients that have recurred has been detected. Therefore, it is essential to identify new biomarkers capable of predicting which subgroup of patients may not respond adequately to therapy or recur. RNA binding proteins are gaining great attention in the cancer field for their capacity to modulate virtually all cancer hallmarks (Pereira et al. 2017). Although they constitute one of the largest protein families in the cell, understanding of their roles in disease is in its infancy (Castello et al. 2013; Gerstberger et al. 2014; Wurth and Gebauer 2015; Hentze et al. 2018). Here we show that the RNA binding protein CIRBP displays pro-oncogenic properties in breast cancer, consistent with previous reports (Chang et al. 2016). Importantly, *CIRBP* transcript levels correlate with poor prognosis in the most frequent luminal A/B breast cancer subtype. To understand the role of CIRBP in luminal breast cancer, we perform the first genome-wide identification of CIRBP targets in cancer, and provide a repository of novel targets with potential relevance in breast oncogenesis. Among them, we identify *CST3* as a functionally relevant target that is down-regulated by CIRBP to promote the tumorigenic properties of breast cancer cells.

The most significant GO terms associated with our list of CIRBP targets are related to the DNA damage response, in agreement with the reported function of CIRBP in this biological process (Yang and Carrier 2001; Yang et al. 2006, 2010; Chen et al. 2018). However, none of the known CIRBP targets are present in our list, indicating alternative ways in which CIRBP can regulate DNA damage. In addition, our list only minimally intersects with transcriptome-wide studies of CIRBP in other biological contexts, suggesting that the functions of CIRBP are context-specific. This is not unusual among RNA binding proteins, which can promote or suppress tumor progression depending on cell type and condition (Pereira et al. 2017). Strikingly, although most known cases of mRNA modulation by CIRBP involve up-regulation of mRNA levels, we find a dominant role of CIRBP in mRNA down-regulation, as most changes after CIRBP depletion include increase in mRNA levels while the opposite is true after CIRBP overexpression. One of the down-regulated targets is *CST3*, encoding for Cystatin C. Cystatins are inhibitors of cysteine proteases, enzymes which play multiple roles in physiological and pathological settings. CST3 is a major inhibitor of cathepsin B, a matrix protease that promotes invasion and escape from immune recognition by degrading antigens and extracellular matrix components (Keppler 2006). In addition to cathepsin-dependent roles, CST3 can suppress tumorigenesis by binding to the TGFβII receptor and acting as a TGFβ antagonist (Sokol et al. 2005). Curiously, a recent report found that breast tumors of *CST3* knockout mice in a breast cancer model were smaller than their wild-type counterparts, suggesting a tumor-promoting role of CST3 (Završnik et al. 2017). However, in this same model *CST3^−/−^* tumors were less aggressive, as they were less capable of metastasizing to the lungs, consistent with a tumor-suppressive role. Our results are consistent with the tumor suppressive capacities of CST3, as CIRBP promotes clonogenicity and anchorage-independent growth by down-regulating CST3 levels. In our model, CST3 seems to act exclusively as a CIRBP mediator, since depletion of CST3 per se has no effect on these traits. Our data are, thus, in agreement with protective roles of CST3 described previously in breast and other cancers such as head and neck, skin, lung, ovarian cancer, and glioma (Konduri et al. 2002; Nishikawa et al. 2004; Strojan et al. 2004; Yu et al. 2010; Mori et al. 2016).

Finally, our network analysis shows that 12 CIRBP targets (*S100A11, MAPK11, STK11, CDT1, HOMER3, TRIM28, RPL28, NCAPH2, SF3A2, SLC25A10, SEZ6L2*, and *MEIS3*) in addition to *CST3* highly intersect with breast cancer genes. One of them, *TRIM28*, is known to promote breast cancer metastasis and is an important indicator of disease progression (Wei et al. 2016; Czerwińska et al. 2017; Damineni et al. 2017). However, to our knowledge, no relation with breast cancer has been reported for the remaining 11 CIRBP targets. It will be interesting to analyze the relevance of these targets for breast cancer progression in future studies.

## MATERIALS AND METHODS

### Constructs

The coding sequence of human CIRBP isoform 1 (172 amino acids) was cloned with a carboxy-terminal FLAG-Tag into the MSCV-PIG (PURO-IRES-GFP) retroviral vector using the Gibson Cloning technology (Gibson et al. 2009) with oligos GibF (5′CTAGGCGCCGGAATTAGATCTCTCAGTGGCCGCCATGGCAT3′) and GibR (5′ TCGTTAACCTCGAGAGATCTTTACTTGTCATCGTCATCCTTGTAATCCTCGTTGTGTGTAGCGTAAC3′). The construct was verified by sequencing.

### Overexpression and depletion

For CIRBP overexpression, MCF-10A cells were incubated with viral particles containing CIRBP-Flag-MSCV-PIG or empty vector (240.000 cells/2 mL viral suspension/well of a six-well plate), and selected with 1 µg/mL puromycin for 3 d before collecting cell extracts. The vector contains EGFP, and thereby all cells express this marker. To obtain viral particles, Phoenix A cells were transfected with the respective constructs, selected with 2.5 µg/mL puromycin after 24 h, the supernatant collected 2 d after puromycin addition and concentrated using Retro-X concentrator (Clontech).

Silencing experiments were performed by transient transfection of siPools (SiTOOLs Biotech) at a concentration of 3 nM using Lipofectamine RNAiMax (Thermo Fisher Scientific) following the manufacturer's protocol. In transfections with two different siPOOLs, 1.5 nM of each were used.

### Proliferation, 3D-growth, clonogenicity, and viability assays

For clonogenicity assays, cells were seeded in triplicates at 2000 cells/well (MCF-7) or 500 cells/well (MCF-10A) in six-well plates, and reverse transfected (i.e., mixed with the transfection mix before plating) with 3 nM siRNA. After 10 d, cells were stained with 0.5% crystal violet (diluted in 25% methanol) for 1 h. Plates were scanned and images quantified using FIJI (Guzmán et al. 2014). Cells were then treated with 10% acetic acid for 30 min to release the dye, and global crystal violet intensity in the supernatant measured at OD_590_.

Proliferation assays were performed by seeding cells after reverse transfection of siPOOLs, followed by cell counting every day for 4–5 d in a Neubauer chamber after trypsinization.

For 3D-growth measurements, cells were seeded at 1000 cells/well in low attachment 96-well plates (Corning) and cultured for 10–14 d with transfection of siPOOLs every 3 d. Cell viability was measured using CellTiter-Glo (Promega) following the recommendations of the vendor.

### Cell extracts and western blot

Cell extracts were prepared by homogenization in RIPA buffer (25 mM Tris-HCL pH 7.6, 150 mM NaCl, 1% NP40, 0.1% SDS, 1% Sodium Deoxycholate and 1× protease inhibitor cocktail from Roche). The cell lysate was spun for 10 min at 13 Krpm and the supernatant was used or snap-frozen and stored at −80°C. The following antibodies were used: anti-CIRBP (Abcam ab94999, 1:500), anti-CST3 (Abcam ab109508, dilution 1:1000), anti-Tubulin (Sigma T9026, 1:5000), anti-FLAG (Sigma F3615, 1:5000), anti-SLC25A10 (Sigma HPA023048, 1:500), anti-TRIM28 (Cell Signaling 4124S, 1:500).

### RNA-immunoprecipitation (RIP)

Cells were washed with PBS and resuspended in lysis buffer (100 mM KCl, 5 mM MgCl_2_, 10 mM Hepes pH 7.0, 0.5% NP40, 1 mM DTT, and RNAsin 40 U/µL), incubated 4 min on ice, snap-frozen in liquid nitrogen, thawed and sonicated in a Bioruptor Diagenode at high level for 10 min with cycles of 30 sec ON/OFF at 4°C. Sonicated cells were spun and the supernatant was precleared with protein A-Dynabeads. On another hand, protein A-Dynabeads were preblocked with 20 µg of tRNA in NET buffer (50 mM Tris-HCl pH 7.5, 150 mM NaCl, 0.1% NP40, 1 mM EDTA) for 15 min at room temperature, washed with NET, and mixed with 8 µg of either anti-CIRBP antibody or anti-rabbit IgG for 1 h at 4°C, washed and resuspended in 100 µL of NET. Eighty microlitres of these beads were mixed with 8 mg cell extract, incubated for 1 h at 4°C, washed four times with five volumes of NET and resuspended in 100 µL of wash buffer. Ten microlitres were used to verify the IP efficiency by western blot. The rest was used for RNA extraction using phenol-chloroform in the presence of 1 µL glycoblue (Ambion). DNase treatment was performed with Turbo DNA-free Kit (Ambion). The same steps were used to isolate RNA from the input lysate.

### RIP-seq and RNA-seq analysis

We performed two replicates per RIP-seq or RNA-seq comparison. Library preparation and sequencing were performed at the CRG Genomics Facility, after assessing RNA quality with Bioanalyzer. The quality of fastq files was assessed with FastQC. Reads were aligned to the human genome (ENSEMBL release 86 for RIP-seq and 88 for RNA-seq) with STAR. The quality of the alignments (BAM files) was assessed with the “rnaseq” module of Qualimap. For RIP-seq, HTSEQ was used to retrieve the number of reads overlapping to each coding gene (option –stranded = reverse), first against the whole annotation, and then only against the three last exons of each transcript. The R/Bioconductor package (DESeq2) was used to calculate the pairwise differential expression of genes between experimental groups.

### Reverse transcription and quantitative PCR

Total RNA was isolated using the mirVana kit (Ambion) and treated with DNase using Turbo DNA-free (Ambion). RNA (800 ng) was reversed transcribed using the RevertAid H Minus First Strand cDNA Synthesis Kit (Thermo Fisher) with random hexamers in a final volume of 20 µL. The resulting cDNA was amplified by Singleplex qPCR using the following Taqman probes: CST3 (4448892-Hs00969174_m1), SLC25A10 (4448892-Hs00201730_m1), TRIM28 (4448892-Hs00232212_m1). Relative gene expression was calculated by comparative ΔΔC_q_ method according to the MIQE guidelines (Bustin et al. 2009).

For the validation shown in Supplemental Figure S1E, immunoprecipitated RNA (typically 100 ng) and 1:100 of input were reverse transcribed with Superscript II using oligo(dT) and random hexamers. The resulting cDNA was amplified using the Power Sybr Green PCR master mix (Applied Biosystems) with the oligonucleotides indicated in Table 1.

**TABLE 1.**
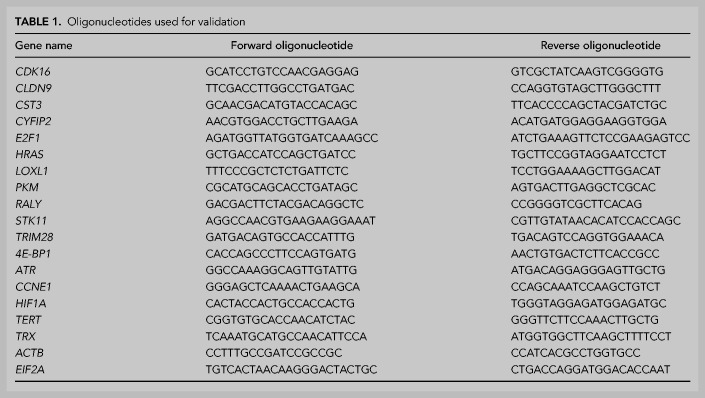
Oligonucleotides used for validation

## SUPPLEMENTAL MATERIAL

Supplemental material is available for this article.
